# Surgical management of mitral valve infective endocarditis with annular abscess and calcification in the setting of a leaking mycotic infrarenal abdominal aortic aneurysm: a case report

**DOI:** 10.1186/s13019-014-0154-0

**Published:** 2014-09-20

**Authors:** Jordan DW Ross, Masashi Ura, Allan Kruger, Jeremy Wright

**Affiliations:** Greenslopes Private Hospital, Newdegate St, Greenslopes, 4120 QLD Australia; Greenslopes Clinical School, University of Queensland, Brisbane, Australia

**Keywords:** Mitral valve endocarditis, Mitral annulus abscess, Mycotic abdominal aortic aneurysm

## Abstract

We present the case of a previously well seventy-four year old male caucasian grazier who presented with mild back pain and was subsequently found to have a large posterior mitral valve leaflet perivalvular abscess associated with mitral annulus calcification and a mycotic infrarenal abdominal aortic aneurysm (AAA) of Staphylococcal origin. He underwent a right axillofemoral bypass with oversewn aorta and a right to left femoral crossover graft, and a subsequent mitral valve repair with decalcification/debridement of the annulus and extensive posterior leaflet reconstruction with pericardium patch. Despite multiple sequelae and an extended intensive care and hospital stay, the patient was discharged home after six months.

**Electronic supplementary material:**

The online version of this article (doi:10.1186/s13019-014-0154-0) contains supplementary material, which is available to authorized users.

## Background

Mitral annulus abscess associated with mitral annulus calcification is a rare but serious form of endocarditis which, though little-reported, imposes a significant increase in technical difficulty of mitral valve repair, has important implications regarding patient selection and operative technique, and carries an increase in morbidity and mortality. We report on the case of a seventy-four year old man with a presentation of posterior mitral valve leaflet abscess in the setting of a Staphylococcus aureus bacteraemia and leaking mycotic abdominal aortic aneurysm.

## Case presentation

### Presentation

A seventy-four year old male Australian grazier presented to a regional base-hospital complaining of one week of back pain and nocturnal fever on the background of three months of malaise and increasing fatigue. He also noted having scratched his arm on a metal grate ten days previously. His medical history was of hypercholesterolaemia, gastro-oesophageal reflux disease, an oesophageal stricture, a previous testicular operation for a benign condition, and degenerative lumbar spine disease. Ethics approval was obtained from the Greenslopes Private Hospital Human Research Ethics Committee prior to beginning the case report.

### Investigation

**Figure 1:**
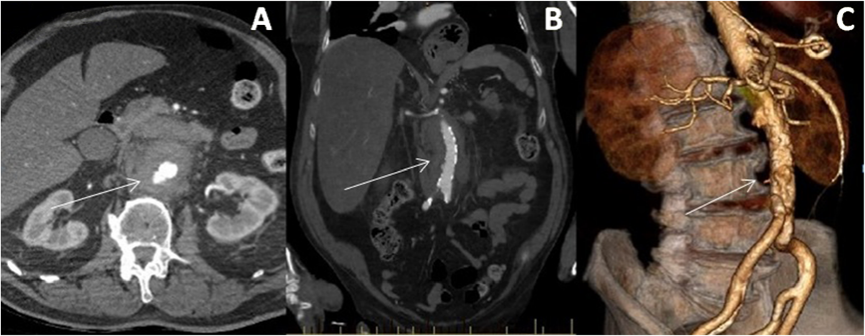
**Imaging of the abdominal aorta prior to surgery. A**. CT showing inflammatory tissue surrounding the leaking abdominal aorta (transverse plane). **B**. Coronal plane showing the abdominal aorta. **C**. Volume-rendered reconstruction of the abdominal aorta.

**Figure 2:**
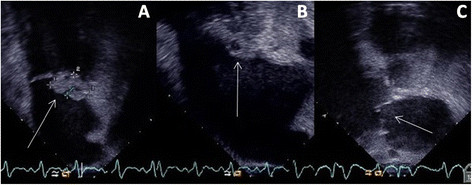
**Echocardiographic imaging of the mitral valve. A**: Transoesophageal echocardiogram showing the large posterior mitral valve leaflet vegetation. **B**: Transoesophageal echocardiogram with arrow indicating leaflet abscess. **C**: Transoesophageal echocardiogram post repair showing a competent mitral valve.

Basic pathology revealed a significant leucocytosis and elevated inflammatory markers. Computed Tomography (CT) and subsequent Magnetic Resonance Imaging (MRI) scans were suspicious for peri-aortitis, and the patient was transferred to our tertiary facility in Brisbane. On arrival a new murmur was noted along with a new expressive dysphasia. Repeat CT revealed a 1.5 cm infrarenal abdominal aortic aneurysm with a surrounding 7.5 cm area of soft tissue abnormality (Figure 1). Echocardiography revealed a large vegetation on the posterior mitral valve leaflet measuring 2.5×1.3 cm with moderate mitral regurgitation and abscess formation within the vegetation and mitral annulus (Figure 2). Left and right ventricular size and systolic function were normal, as were the other cardiac valves.

### Initial treatment

The patient initially underwent an excision and oversew of his infrarenal aorta with a right axillofemoral bypass and right to left bifemoral crossover grafts. At time of operation, the abdominal aortic wall consisted of friable connective tissue with a large haematoma and pus. The excised tissue later grew Staphylococcus aureus sensitive to flucloxacillin.

**Figure 3:**
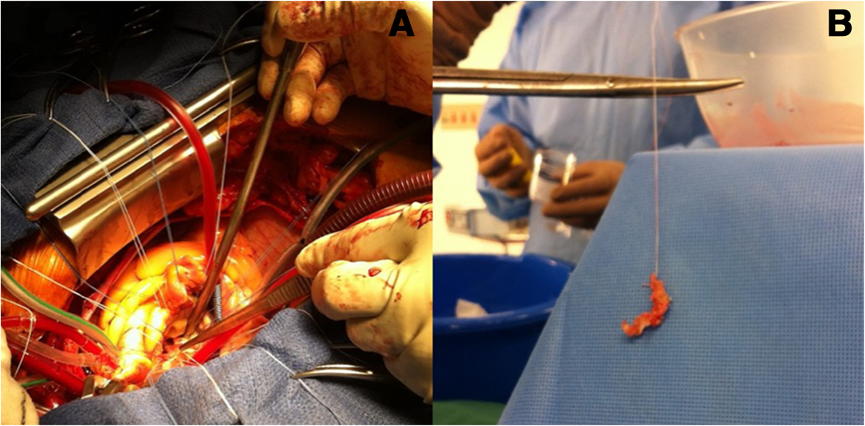
**Photographs of the mitral valve and resected calcification. A**: Intraoperative photograph of the mitral valve. **B**: The resected specimen.

Post-operatively, the patient developed multiple signs of distal embolisation including upper and lower limb digital splinter haemorrhages, cerebellar and splenic infarctions. Decision was made to attempt repair of the mitral valve, which was performed via median sternotomy four days after the initial admission and repair of the AAA. Aortic and bicaval cannulation was employed with hypothermia to thirty-two degrees. The mitral valve was accessed through Sondergaard’s groove. Visual inspection of the valve revealed a gross distortion of the posterior leaflet with a large vegetation and P1/P2 abscess cavity in the setting of circumferential mitral annulus calcification (Figure 3) with the posterior annulus most severely affected.

The diseased valve leaflet was detached, the adjacent abscess debrided, and the mitral annulus extensively decalcified. The defect in the annulus was repaired with a pericardial patch and the posterior leaflet reattached to the neo-annulus (made of pericardium and left atrium). The remaining leaflet tissue was augmented with pericardium and a 32 mm Physio II (Carpentier-Edwards) annuloplasty ring was employed.

### Complications and subsequent treatment

The patient was treated in the Intensive Care Unit for five weeks, during which time he was treated for multiple complications including atrial fibrillation with rapid ventricular response, left ventricular failure, bilateral pleural effusions, acute kidney injury, drug-induced hepatitis, coagulopathy, Pseudomonas pneumonia, upper limb deep vein thrombosis and Clostridium difficile colitis. Several toe amputations were performed due to necrotic digits after septic embolisation. A sternal sinus also developed requiring two debridements and extensive antibiotic treatment. Follow up echocardiograms showed a competent mitral valve with no evidence of endocarditis (Figure 2C). Effective orifice area was 2.6 cm^2^ with no evidence of an elevated trans-mitral gradient. Tissue culture confirmed Staphylococcus aureus and subsequent blood cultures while on antibiotics were shown to be negative. The patient received a total of eight weeks of intravenous flucloxacillin. The patient was discharged from the rehabilitation unit six months after his admission date.

## Discussion

We report a case of Staphylococcal mitral valve abscess and abdominal aortic aneurysm. The presentation of periaortitis with mitral valve infective endocarditis and annular abscess with calcification presents important diagnostic and treatment considerations.

It has been shown that the well understood morbidity and mortality benefit conveyed by mitral valve repair compared to replacement in the setting of degenerative mitral valve disease is also relevant in the presence of mitral disease caused by infective endocarditis [1]-[5]. A metaanalysis of data comparing repair and replacement in endocarditis, performed by Feringa and colleagues [3], showed lower in- hospital and long term mortality, less frequent repeat surgery, fewer cases of recurrent endocarditis and fewer cerebrovascular events in those patients who underwent valve repair. For these reasons we advocate reasonable persistence in achieving valve repair, placing importance in the complete removal of infected tissue. Data from De Kerchove and colleagues reinforces our view on the satisfactory durability of patch repair techniques [1].

Factors variably reported as predictors for repair failure are acute surgery, heart failure, uncontrolled sepsis, renal failure, paravalvular abscess, and mitral annulus calcification [1],[3],[6],[7]. Severe annulus calcification has been known to increase the technical difficulty of mitral valve repair, and impose significant risk of complications from valve replacement [8]. Several studies have now established feasibility of mitral valve repair in the setting of annulus calcification, and in the data of d’Alessandro and colleagues, reported a five-year survival benefit when comparing repair with replacement, with the main determinant of repair feasibility being extent of calcification [6],[7]. Valve repair was possible in 68% of patients with an in-hospital mortality (14%) that was increased in patients with infective endocarditis [6].

Timing and staged order of the surgical procedures is an important point of consideration. The clinical severity and immediate sequelae of an untreated leaking AAA dictates urgent repair of this issue in the first instance. It is important to note that elimination of non-cardiac infective sources prior to valve surgery is an important principle which reduces the risk of recurrent endocarditis and is particularly important if valve replacement is required. This has been shown in the case of mitral valve infective endocarditis with splenic abscess. Shang and colleagues [4] have shown that half of mitral valve endocarditis patients had septic embolic neurological findings on CT head (most being symptomatic), and have shown that low rates of morbidity and mortality are possible with early operation (within four days of admission). It is reasonable to suggest that the longer an already embolising mitral valve abscess is left unrepaired, the more frequent and severe the likely embolic phenomenon will be, especially in cases of large valve vegetations/abscesses (as in our case). This risk is balanced by the increased risk of intra-operative cerebral haemorrhage in the setting of recent cerebral infarction. Furthermore, we agree that early operation will minimise valve destruction and improve the haemodynamics compromised by mitral regurgitation [4]. For this reason we elected to attempt valve repair shortly after the patient was haemodynamically stabilised by resuscitation and repair of the leaking AAA, in the knowledge that if valve repair was not possible, the early operation may carry increased risk of prosthetic valve endocarditis. Full heparinisation and cardiopulmonary bypass imposed significant risk of bleeding to simultaneous AAA and mitral valve repairs. A temporising endovascular procedure to the AAA was considered, but definitive surgical repair and abscess decontamination was undertaken in the first instance due to the inevitability of stent graft infection, as well as the high risk of renal failure due to stent graft obstruction of the closely located renal vessels.

It was important this patient was treated in a specialist centre (tertiary facility) by a multidisciplinary team from surgical, intensive care, anaesthetic, cardiology and allied health departments; and we advocate a tailored approach to complex cases of mitral valve endocarditis.

## Conclusion

Posterior mitral valve leaflet abscess and a leaking mycotic abdominal aortic aneurysm poses a difficult diagnostic and treatment dilemma of cause and effect. Repair should be attempted in all cases of mitral valve endocarditis meeting long-established indications for surgical intervention, providing that all infected tissue can be excised and a minimally regurgitant valve reconstructed. Elimination of extracardiac sources with staged heart surgery of infection is an important consideration in valve repair/replacement.

## Consent

Written informed consent was obtained from the patient for publication of this Case report and accompanying images. A copy of the written consent is available for review by the Editor-in-Chief of this journal.

## Authors’ contributions

JR drafted the manuscript. MU edited and revised the manuscript for important intellectual content. All authors read and approved the final manuscript.

## Authors’ information

JR is a cardiothoracic and vascular surgical resident at Greenslopes Private Hospital.

MU is a consultant cardiothoracic surgeon at Greenslopes Private Hospital, and was the operating cardiac surgeon in the above case.

JW is a cardiologist at Greenslopes Private Hospital, and was the operating cardiologist in the above case.

AK is a vascular surgeon at Greenslopes Private Hospital, and was the operating vascular surgeon in the above case.

## Acknowledgements

None.

## Authors’ original submitted files for images

Below are the links to the authors’ original submitted files for images. Authors’ original file for figure 1 Authors’ original file for figure 2 Authors’ original file for figure 3
