# Supplementary figures and images for: Surgical management of mitral valve infective endocarditis with annular abscess and calcification in the setting of a leaking mycotic infrarenal abdominal aortic aneurysm: a case report
Source: J Cardiothorac Surg. 2014 Sep 20;9:154. doi: 10.1186/s13019-014-0154-0 (PMC4181699; doi:10.1186/s13019-014-0154-0)

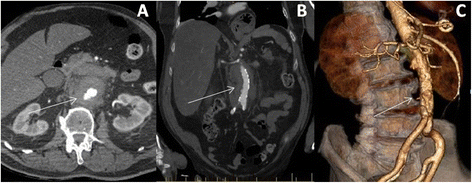

Supplement: Supplementary file 1 — Authors’ original file for figure 1 [file 13019_2014_154_MOESM1_ESM.gif]

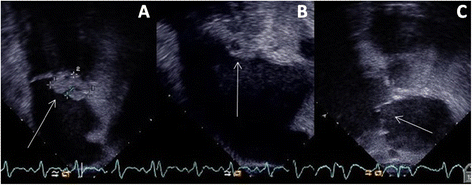

Supplement: Supplementary file 2 — Authors’ original file for figure 2 [file 13019_2014_154_MOESM2_ESM.gif]

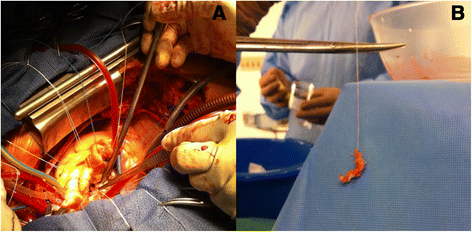

Supplement: Supplementary file 3 — Authors’ original file for figure 3 [file 13019_2014_154_MOESM3_ESM.gif]
